# Modeling and Dynamics of the Inward-Facing State of a Na^+^/Cl^−^ Dependent Neurotransmitter Transporter Homologue

**DOI:** 10.1371/journal.pcbi.1000905

**Published:** 2010-08-26

**Authors:** Saher Afshan Shaikh, Emad Tajkhorshid

**Affiliations:** 1 Department of Biochemistry and Beckman Institute, University of Illinois at Urbana-Champaign, Urbana, Illinois, United States of America; 2 College of Medicine and Center for Biophysics and Computational Biology, University of Illinois at Urbana-Champaign, Urbana, Illinois, United States of America; Weill Medical College of Cornell University, United States of America

## Abstract

The leucine transporter (LeuT) has recently commanded exceptional attention due mainly to two distinctions; it provides the only crystal structures available for a protein homologous to the pharmacologically relevant neurotransmitter: sodium symporters (NSS), and, it exhibits a hallmark 5-TM inverted repeat (“LeuT-fold”), a fold recently discovered to also exist in several secondary transporter families, underscoring its general role in transporter function. Constructing the transport cycle of “LeuT-fold” transporters requires detailed structural and dynamic descriptions of the outward-facing (OF) and inward-facing (IF) states, as well as the intermediate states. To this end, we have modeled the structurally unknown IF state of LeuT, based on the known crystal structures of the OF state of LeuT and the IF state of vSGLT, a “LeuT-fold” transporter. The detailed methodology developed for the study combines structure-based alignment, threading, targeted MD and equilibrium MD, and can be applied to other proteins. The resulting IF-state models maintain the secondary structural features of LeuT. Water penetration and solvent accessibility calculations show that TM1, TM3, TM6 and TM8 line the substrate binding/unbinding pathway with TM10 and its pseudosymmetric partner, TM5, participating in the extracellular and intracellular halves of the lumen, respectively. We report conformational hotspots where notable changes in interactions occur between the IF and OF states. We observe Na2 exiting the LeuT-substrate- 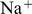 complex in the IF state, mainly due to TM1 bending. Inducing a transition in only one of the two pseudosymmetric domains, while allowing the second to respond dynamically, is found to be sufficient to induce the formation of the IF state. We also propose that TM2 and TM7 may be facilitators of TM1 and TM6 motion. Thus, this study not only presents a novel modeling methodology applied to obtain the IF state of LeuT, but also describes structural elements involved in a possibly general transport mechanism in transporters adopting the “LeuT-fold”.

## Author Summary

Elucidating the mechanism of active transport across the membrane is relevant not only to the understanding of physiological processes but also to the rational design of drugs that modulate these processes. In the cell membrane, specialized proteins known as secondary transporters utilize the energy stored in the electrochemical gradient of ionic species across the membrane in order to carry out active transport. The leucine transporter is such a secondary transporter, with the unique distinction of being homologous to clinically relevant neurotransmitter transporters, and also similar in architecture to several other secondary transporters that are unrelated by sequence. This similarity establishes the significance of the typical “LeuT-fold” in secondary transporter function. In this study, we set forth to model and study the dynamics of LeuT in an alternative conformational state of the transport cycle, for which no crystal structure is known. A novel methodology is developed, yielding models of the inward-facing state of LeuT. We discuss several key features of this state, including structural elements and interactions that participate in the transition to this state. The study thus enhances the understanding of the transport mechanism of several families of “LeuT-fold” transporters, most including known and putative drug targets.

## Introduction

Leucine transporter (LeuT) is a bacterial amino acid transporter, homologous to 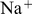/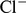 dependent neurotransmitter transporters of the solute carrier 6 (SLC6) family, which is also known as the neurotransmitter:sodium symporter (NSS) family [1]–[3]. This family of transporters includes several clinically relevant drug targets for neurological conditions such as depression, ADHD, anxiety and drug abuse; e.g., transporters for serotonin (SERT), dopamine (DAT), norepinephrine (NET) and 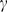-aminobutyric acid (GAT-1) [4]–[6]. These neurotransmitter transporters are functionally well-characterized, however, crystal structures for this family are only available for LeuT [2], [7]–[11], which thus provide critical structural information for the NSS family.

**Figure 1:**
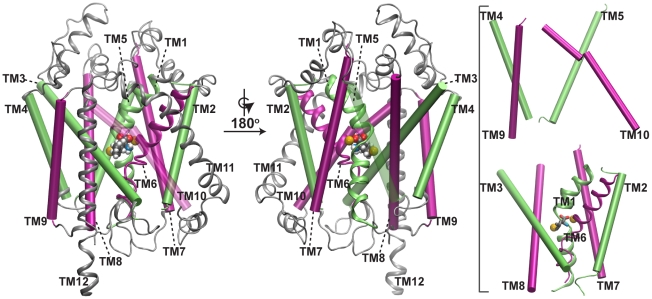
The crystal structure of LeuT. Views of the LeuT structure as would be seen along the plane of the lipid bilayer, oriented such that the upper side represents the extracellular half. Left: The pseudosymmetric 5+5 repeat is shown in color (green: first repeat, TM1 to TM5; pink: second repeat, TM6 to TM10) with the two broken helices in the core, TM1 and TM6, shown in ribbon representation. The rest of the protein, including TM11 and TM12 are in gray. Bound 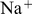 ions are in yellow, and the substrate, leucine, is colored by element name (H: white, C: gray, N: blue, O: red). TM10 is transparent for clarity. Center: A 180-rotated view of the structure is shown. TM5 is transparent for clarity. Right: The outer scaffolding helices, TM4, TM5, TM9, and TM10, are shown separated (above) from TM1, TM2, TM3, TM6, TM7, and TM8 (below) for clarity.

The first structure of LeuT [2] (Fig. 1) presented a high resolution (1.65 Å) picture to complement several earlier functional studies on NSS family members that revealed residues involved in gating, and in binding and translocation of the ions and the substrate/inhibitor [12]–[16]. This structure has also served as an important model for the interpretation of structural consequences of mutagenesis and accessibility experiments on NSS members [17]–[21]. Multiple crystal structures of LeuT bound with the substrate and inhibitors have been also reported [2], [7]–[11]. Notable computational studies taking advantage of these structures include the prediction of the 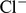 binding site in SERT [22] and GAT-1 [23], the prediction of a model for the inward-facing conformation of LeuT [20], prediction and confirmation of a second substrate binding site in the extracellular lumen [10], [24], [25], and computational simulations designed to probe substrate/ion binding [26]–[28] and the putative transport mechanism [24], [28].

LeuT is composed of 12 transmembrane (TM) helices, of which helices 1–5 and 6–10 form two pseudosymmetric domains, arranged as an inverted repeat (Fig. 1). The crystal structures characterized the position of the bound substrate and two 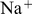 ions, consistent with the 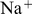 -dependent function of the NSS family (Fig. 1). In the extracellular (EC) lumen the substrate is protected from the EC solution by two pairs of conserved residues, Y108∶F253 and R30∶D404. This state, where the lumen is open outwards, but the substrate is “occluded” by the lining residues, can be referred to as the outward-facing-occluded (OF-occ) state.

LeuT is proposed to function according to the “alternating access” model [29], where the transporter alternates between the outward (extracellular) facing-open (OF-o) and inward (intracellular) facing-open (IF-o) states, going through intermediate OF-occ and inward-facing-occluded (IF-occ) states. The occluded states refer to structures where the substrate is bound but not directly accessible to the extracellular or intracellular solutions. Though several crystal structures of LeuT have been reported, they have only been able to capture LeuT in its outward-facing (OF-o and OF-occ) conformational states.

Based on the internal symmetry of LeuT, a model for the inward-facing (IF) state has been reported [20]. In this model, the structures of the two pseudosymmetric domains of LeuT were exchanged, resulting in a putative IF state. The model was further refined using accessibility studies on SERT [20]. While this model provided the first structural description of a possible IF state, it being a static model, the structural elements involved in the OF-to-IF transition could only be deduced from a comparison of the crystal OF and modeled IF states. Based on these states, the transport mechanism is described in terms of rigid body motions of the TM1-TM2-TM6-TM7 bundle in LeuT [20], [30]. In an independent simulation study, the substrate was pulled along the EC and IC directions to investigate the substrate transport pathways [24]. The EC-side pulling revealed a second substrate binding site (S2) which was supported by multiple experimental studies [10], [24], [25]. The IC-side pulling, though not capturing large conformational changes involved in the OF-to-IF transition of LeuT, revealed some elements of the structural changes that might accompany the transition, e.g., hydrogen bond and salt-bridge dissociation and rearrangements. Both these studies thus provide vital information about the possible structural changes involved in formation of the IF state in LeuT. However, in the absence of sufficient information about IF state dynamics and that connecting the transition between the OF and IF states, the transport mechanism remains only partially understood. Moreover, recent reports of “LeuT-fold” transporters in the IF state, opens up new opportunities for building the IF state models. Keeping this in view, the present study was designed to model and describe the dynamics of the IF state, by inducing the OF-to-IF transition using structural information from existing crystal structures.

The inverted-repeat pseudosymmetry was an unprecedented feature of the LeuT structure when it was first reported [2]. Subsequent discovery of this typical fold with 5+5 inverted repeats in several crystal structures from other families of transporters, namely, vSGLT [31], Mhp1 [32], [33], BetP [34], AdiC [35], [36], ApcT [37] and CaiT [38] has established strong functional relevance of this fold in secondary transporter function [39], [40].

The sodium galactose transporter, vSGLT, belonging to the solute sodium symporter (SSS) family, exhibits the “LeuT-fold”, and its structure is reported in the IF state [31]. Here, we use this structure to generate a model for the IF state of LeuT. Since vSGLT has very low sequence similarity to LeuT, standard homology modeling techniques were inapplicable. Hence we devised a combination of techniques, including structure-based alignment, threading, targeted molecular dynamics (TMD) and equilibrium MD, to exploit the architectural similarity between LeuT and vSGLT in the core “LeuT-fold”, for modeling. The methodology allows generation of models and a description of a possible IF state, also providing hints about the OF-to-IF transition. The final models retain the secondary structural features of LeuT and the substrate binding sites, while adopting an IF state. We have also monitored the dynamics of the IF state and the OF-to-IF transition of LeuT, describing the main structural elements of LeuT involved in this transition and related changes in interactions.

We seek answers to some questions pertinent, specifically, to the description of the mechanism of several known as well as proposed drug targets, and generally, to enhance understanding of the dynamics of transporter function: What does the IF state of LeuT look like, and which elements form the substrate and 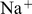 release pathway? Which structural elements are involved in the alternation of LeuT between the OF and IF states? Does the 5+5 symmetry have direct relevance to the function of the transporter, and do the inverted pseudosymmetric partners also show symmetry in function? This study allows us to answer, or reveal hints to the answers of these, and several related questions.

## Methods

The main aim of this study is to describe the IF state of LeuT. To this end, we generated IF models of LeuT based on the known crystal structure of vSGLT in the IF-occ state, incorporating a dynamic OF-to-IF transition in the process.

We evolved a unique methodology to tackle the challenges arising in achieving these aims. The model was generated using a combined approach employing structure-based alignment, threading, and targeted molecular dynamics (TMDs). In TMD, a starting structure is guided towards adopting the conformation of a target structure, during a molecular dynamics (MD) simulation [41]. We used the TMD algorithm as implemented in the NAMD package [42]. Here, a subset of atoms chosen from the simulation system are guided towards a pre-defined target conformation by means of targeting forces. The forces are calculated at each time step from the gradient of the potential given by
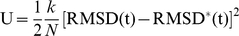
where k is the applied force constant, N is the number of atoms targeted, RMSD(t) is the root mean square deviation between the current and the target structures calculated at each time step, and, 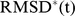 changes linearly with time from the initial RMSD between the current and target structure to the targeted final RMSD. The targeted final RMSD was defined as 0 in all TMD simulations performed in this study. The TMD terminates after it has completed the pre-defined number of steps, which is related to the total time of the simulation (1 ns and 50 ns for different sets of TMDs in this study, as discussed later).

**Figure 2:**
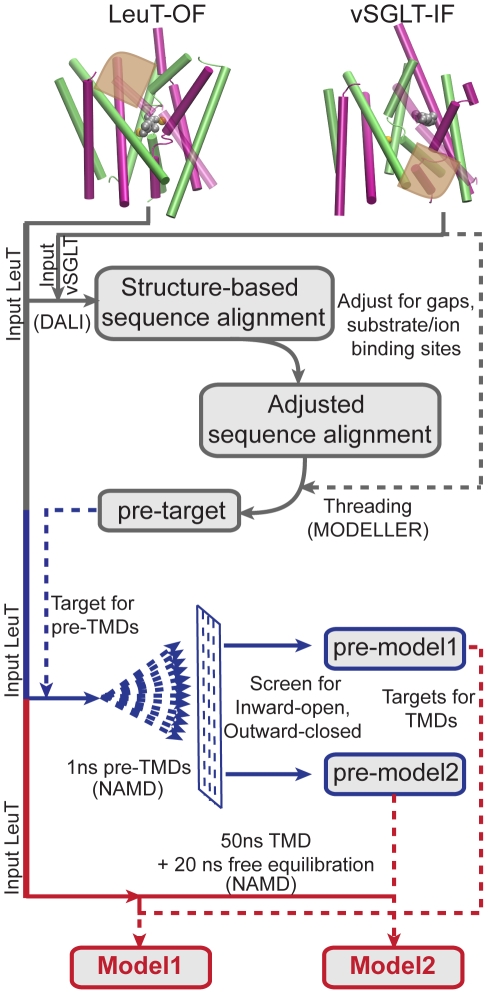
Schematic of the modeling methodology. The combined approach adopted for generation of the inward-facing model is presented (see Methods). The location of the EC and IC halves of the lumen in the LeuT and vSGLT structures, respectively, are marked in brown. The initial phase of structural alignment and threading (gray), the pre-TMD phase (blue), and the final TMD phase (red) resulting in the two models discussed in this study, are illustrated. Dotted lines are used for structural input in addition to the starting LeuT structure.

In our modeling methodology, we use TMD at two distinct stages of the model generation - short (1 ns) TMDs in the initial stage to generate “pre-models”, and, extended (50 ns) TMDs for obtaining the final models using the pre-models as targets. To distinguish between these two sets of TMDs and their purposes, the short TMDs are henceforth referred to as “pre-TMDs” (pre-model generation TMDs) and the two long TMDs are simply referred to as “TMD”. The methods described below are accordingly divided into two sections- “pre-model generation” and “Model generation”. The techniques involved, including target design, system setup and simulation protocols, are described here. The methodology is presented schematically in Fig. 2.

### “Pre-model” generation

“Pre-models” of LeuT, to be used as targets for the final TMDs, were generated by combining information from the crystal structures of OF-occ LeuT (PDB code: 2A65) [2] and IF-occ vSGLT (PDB code: 3DH4) [31], in the following manner.

#### Design of the inward-facing LeuT “pre-target”

**Figure 3:**
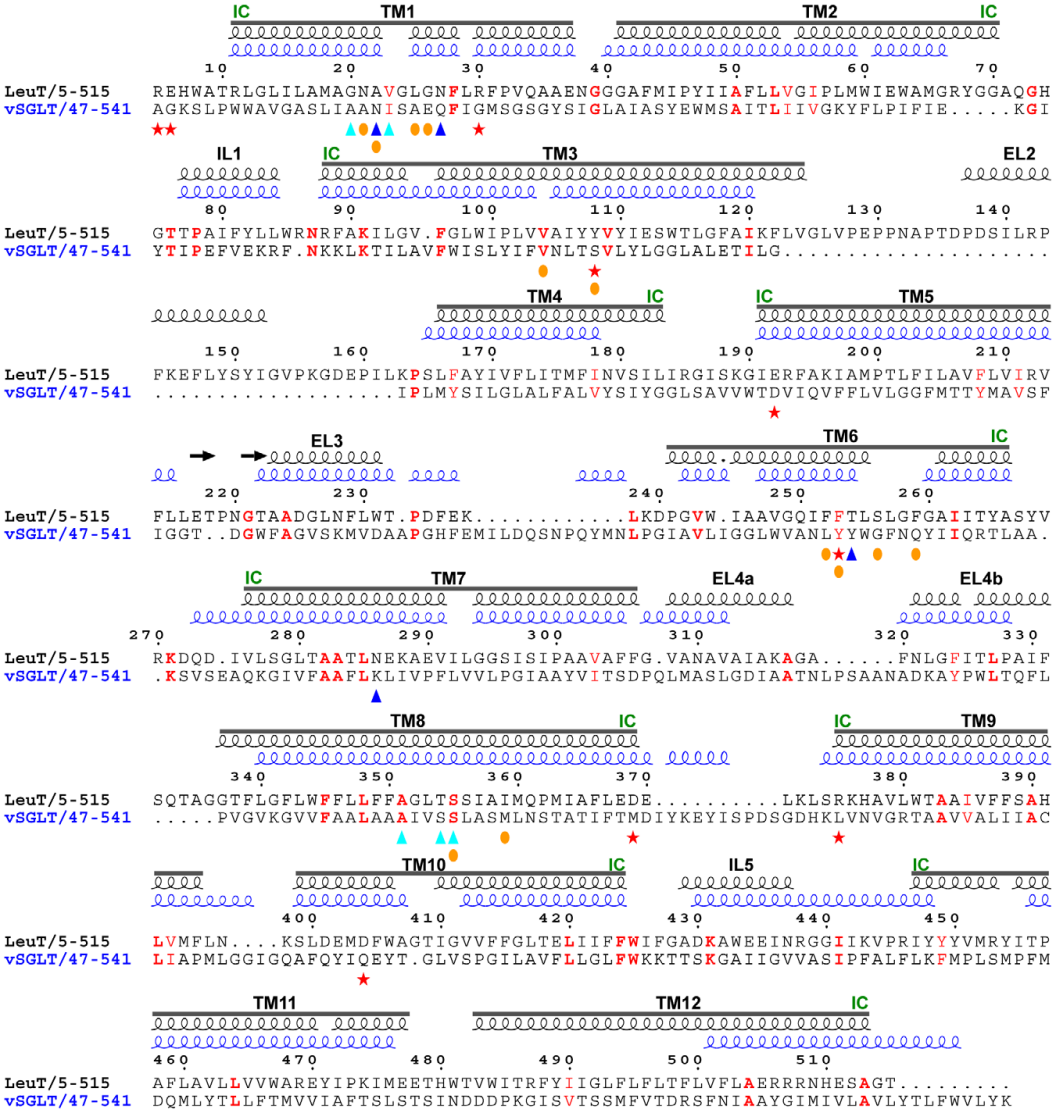
The LeuT-vSGLT sequence alignment adopted for “pre-target” generation. The sequences corresponding to TM1-TM10 along with intermediate loops (EL: extracellular loops, IL: intracellular loops) are indicated. The intracellular ends of the helices are marked as “IC”. Identical and similar residues between the two sequences are colored red, with the former in bold. LeuT residues that bind Na1 (blue triangles), Na2 (cyan triangles) and substrate (orange circles) are marked. Also marked are important residues (red stars), determined from this and previous studies, involved in gating and Na2 release.

LeuT and vSGLT share less than 10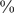 sequence identity in the LeuT-fold core region, thus homology modeling could not be applied directly, and a structure-based modeling approach was adopted. Structure-based sequence alignment of LeuT upon vSGLT was performed using DaliLite [43]. The resulting sequence alignment was initially used directly as input for threading with Modeller [44], which generated structures for LeuT. However, the structures obtained from this alignment turned out unsatisfactory due to the variation in lengths of corresponding secondary structures (helices and loops) between LeuT and vSGLT - the substrate, Na1, and Na2 binding sites were not fully preserved, and a large loop between TM3 and TM4 was incorrectly modeled. Hence, two modifications were made. First, in order to maintain the relative orientation of side chains forming the substrate, Na1, and Na2 binding sites in the LeuT crystal structure, the alignment was manually adjusted. These adjustments included removal of gaps in TM1-TM3 in regions where continuous TM helices are present in the LeuT crystal structure, and modification of the starting positions of helices TM4-TM7 to conform better to the LeuT crystal structure. Second, the LeuT sequence for the TM3-to-TM4 loop was added to the alignment, such that this loop is modeled using the LeuT structure while the rest of the structure is modeled using vSGLT. The modified alignment thus obtained (Fig. 3) was used as input for threading with Modeller. Five models were obtained and evaluated, and the model with the best energy score was chosen. This structure was reported to have fair quality by PROCHECK [45], with 90.9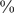 residues in the core region and 0.7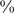 in the disallowed region. A preliminary structure of IF LeuT based on the vSGLT structure was thus obtained.

While this structure exhibits an IF state and is improved over the preliminary alignment-based model, it still suffers from incorrect modeling of regions which do not have a direct equivalent in the vSGLT structure, and most secondary structural elements apart from TM1-TM10 are not well-preserved. Inclusion of all these regions from the LeuT structure into the modeling, as we did for the TM3-to-TM4 loop, is not advisable since this could introduce errors and unfavorable conformations due to their incompatibility with the vSGLT structure. To eliminate these problems associated with the model, we decided to use it merely as a target (henceforth referred to as “pre-target”) to guide the modeling rather than using it directly (Fig. 2). The superimposition of TM1-TM10 of the pre-target on the LeuT crystal structure is presented in Figure S1 (Supporting Information). We thus combined the above approach of structure-based alignment and threading, with the technique of TMD. Since TMD directs the original OF-occ LeuT structure towards the target structure, it allows incorporation of all secondary structural elements apart from TM1-TM10, e.g. the TM3-to-TM4 loop, EL4 and IL1 loops, TM11, and TM12, while also allowing them to respond to the conformational change being induced.

#### System setup and simulation protocol for pre-TMDs

The crystal structure of LeuT (PDB Code 2A65) [2], embedded in lipid membrane and equilibrated for 10 ns in the presence of water and ions, was taken as a starting point for the pre-TMD simulations. It is not known whether the functional state of LeuT is monomeric or dimeric, hence we chose to simulate the system as reported in the crystal structure (dimeric state) [2]. The added advantage provided by this choice is that the dimeric form of LeuT in the crystal structure represents a plausible arrangement of the monomers that can be studied with a smaller simulation system than two separate monomeric systems, hence allowing more efficient sampling.

Considering that both monomeric units may not necessarily undergo the outward to inward transition at the same time, only one monomer was subjected to TMD (monomer A) while the second monomer (monomer B) undergoes free MD. This is consistent with experimental reports of simultaneous transport and counter-transport by oligomeric subunits of monoamine transporters [46], and functional independence of subunits in the oligomeric structure of rGAT1 [47]. Moreover, we do not observe any appreciable structural change at the helices involved in the dimer interface (TM12 and TM9), or in monomer B as a result of TMD in monomer A, as evident from the structural analyses presented later. Hence we consider monomer B simulations as a control. It may be noted, however, that we do not consider these observations as a proof of conformational uncoupling between the LeuT monomers; it is possible that on longer time scales and during other stages of the transport cycle, such proximity among monomers may hold functional relevance.

A detailed description of the system setup and protocol for the initial 10-ns equilibration of the LeuT dimer is presented in [28]. In brief, the following steps were involved. Missing residues (N133, A134) in the crystal structure were modeled. Based on pKa calculations, E112, E287, E419, H74 and H391 were modeled in their protonated form. Bound substrate and 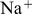 ions (Na1 and Na2) were retained, with the substrate, leucine, modeled as a zwitterion. The dimer was inserted in a POPE membrane bilayer (140 Å
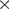
125 Å) and solvated. 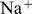 and 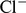 ions were added to neutralize the system and to obtain an ionic concentration of 0.2 M.

All simulations were performed using NAMD [42], adopting periodic boundary conditions. The CHARMM27 force field [48] with CMAP corrections [49] was employed for describing protein, lipids and ions while the TIP3P model was used for water [50]. Short-range non-bonded interactions were calculated using a cutoff distance of 12 Å, and long-range electrostatic interactions were calculated using the particle mesh Ewald (PME) method [51]. For all simulations, a constant temperature of 310 K was maintained by employing Langevin dynamics with a damping coefficient of 0.5 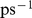 while the Langevin piston method [52], [53] was employed to maintain a constant pressure of 1.0 atm with a piston period of 100 fs. The area of the lipid bilayer was maintained constant. Details of the initial steps of equilibration are described in [28]. Following these, 10 ns of equilibrium simulations were performed. The final equilibrated structure obtained from these simulations was employed as the starting structure in pre-TMD simulations.

A series of pre-TMD simulations were performed, wherein the expected output was an IF conformation. These simulations serve as a fast method of screening for inward-open, outward-closed structures to be used as targets for the final TMD simulations. During the pre-TMD simulations, either all ten TM helices of the LeuT fold, or several subsets of these helices from monomer A were included in the targeted set. Targeting forces were applied only to C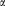 atoms of residues of the targeted helices for 1 ns each, using a force constant 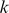 of 200 kcal/mol/Å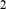 (effective force constant 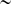
0.5 to 1.5 kcal/mol/Å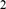 on each C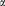 atom, depending on the number of targeted C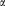 atoms). The rest of the system, i.e., all the side chains, backbone amide bonds, the loops, TM11 and TM12 of monomer A, the complete monomer B, lipids, water and ions undergo free MD.

While opening at the cytoplasmic side occurred in all the pre-TMD simulations, some also showed an unexpected opening on the extracellular (EC) side. We found that this occurred due to a large difference in the orientation of the EC halves of TM1 and TM6, between the vSGLT and LeuT structures. In LeuT, the EC halves of TM1 and TM6 are nearly parallel, while in vSGLT, they are “V-shaped”, mainly due to the TM6 orientation. This appears to be due to the substrate position in vSGLT, which sits between TM1 and TM6 near the base of the “V”, while in LeuT, the substrate binds at the ends of the two broken EC halves. Exclusion of TM6 from the targeted set was found to eliminate this apparent artifact and allowed TM1 and TM6 to retain a parallel orientation in the EC side. We thus observed two cases where inward-opening is accompanied along with outward-closure; when forces are applied to only one half of the LeuT fold (TM1 to TM5), and when the broken helices TM1 and TM6 were excluded from TMD application (i.e., only TM2 to TM5, and TM7 to TM10 are targeted). The final structures obtained from these two pre-TMD simulations thus provide two “pre-models”, respectively. Both pre-models were reported to have good structure quality by PROCHECK [45], with pre-model1 (TM1 to TM5 targeted) and pre-model2 (TM2 to TM5 and TM7 to TM10 targeted) showing 95.0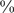 and 94.5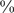 residues in the core region, and 0.2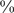 and 0.0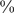 in the disallowed region, respectively. Both pre-models preserve the secondary structural elements and substrate/ion binding sites of LeuT, and represent a plausible IF conformation. These pre-models were used as targets in the next stage of extended TMD simulations i.e., in model generation (Fig. 2).

### Model generation

TMDs were performed to obtain the final IF models, by inducing an OF-to-IF transition guided by the pre-models. The equilibrated LeuT dimer obtained after 10 ns simulation (discussed in the previous section) was again used as the starting structure. The two IF pre-models were used as the targets (Fig. 2). The TMDs were applied to monomer A of LeuT, while monomer B underwent free MD.

Corresponding to the two pre-models being used as targets, two TMDs were performed: TMD-1, where forces are applied to only one half of the LeuT fold (TM1 to TM5), and, TMD-2, where helices TM1 and TM6 were excluded from force application. In TMD-1, targeting forces were applied with a force constant of 141 kcal/mol/Å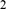, on 141 C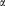 in monomer A, effectively resulting in a force constant of 1 kcal/mol/Å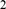 on each C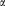 in the targeted set. Similarly, in TMD-2, a force constant of 228 kcal/mol/Å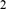, was applied on 228 C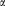 in monomer A. Both the TMDs were 50 ns long. After completion, each TMD was followed with 20 ns of free equilibrium MD with no constraints or additional forces. The two 70 ns simulations (TMD followed by equilibration) are referred to as 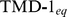 and 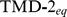, respectively. Other simulation setup and protocol details were the same as discussed in the previous section. The final structures obtained after 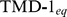 and 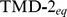 represent the IF models, which we refer to as “Model1” and “Model2”, respectively. The PDB coordinates of Model1 and Model2 have been made available as Dataset S1 and Dataset S2 (Supporting Information), and movies of 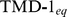 and 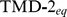, are available as Video S1 and Video S2 (Supporting Information), respectively. Since both models represent the IF state, but differ in conformation (details in Results and Discussion), we consider them as representing two possible snapshots of the IF state, and include both in all subsequent analyses. Additionally, the monomer undergoing free MD (monomer B) is also included in some analyses, as a control system subjected to the same conditions as monomer A, except for the TMD forces.

### Analysis

**Figure 4:**
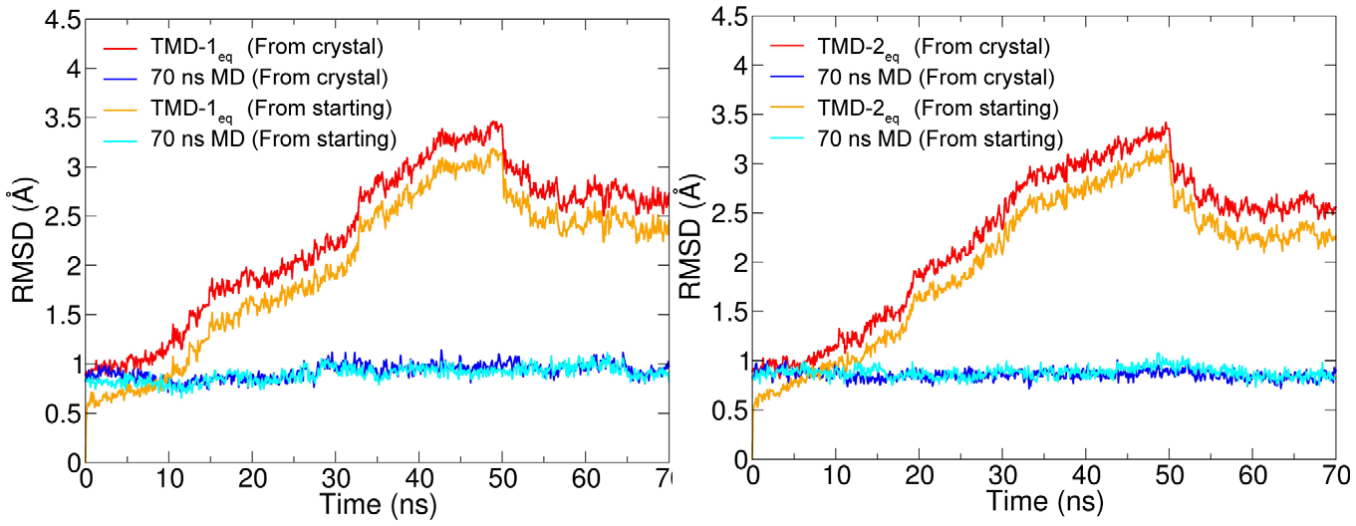
RMSD variation in 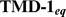 and 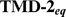. Root mean square deviations (RMSDs) of C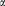 positions from the crystal structure (red, blue) and from the starting (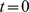 ns) structure (orange, cyan) during the 50 ns TMD+20 ns equilibration in monomer A (red, orange), compared to those in 70 ns free MD of monomer B (blue, cyan). The starting structure (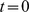 ns) of these simulations corresponds to a structure obtained after 10 ns of equilibration performed previously (see Methods). For both 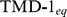 (left) and 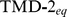 (right), the structures relax to a conformation distinctly different from the reference OF-occ structures.

Root mean square deviations (RMSD) were calculated for C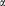 positions of helices TM1 to TM10, compared to the reference structures, along the 50 ns TMD+20 ns equilibration in monomer A as well as 70 ns free equilibration in monomer B in both systems (Fig. 4). The reference structures were the crystal structure, and monomer A from the 10 ns equilibrated structure used as starting structure for TMD.

**Figure 5:**
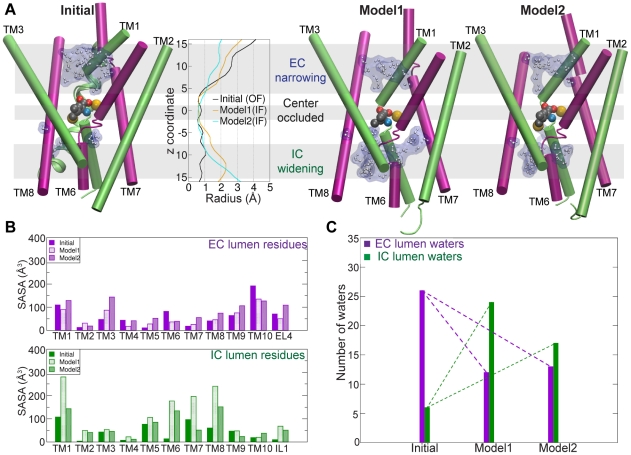
Water accessibility of the IF state models. A. Comparison of TM domains and water penetration in the initial structure input to TMD (left), and in Model1 and Model2 (right). Water molecules (white atoms, blue surface) in the lumen are shown. TM4, TM5, TM9, and TM10 are hidden for clarity. The radius profile of the lumen (center) shows EC narrowing and IC widening in both Model1 (orange curve, radii averaged over 1 ns) and Model2 (cyan curve, radii averaged over 1 ns) as compared to the initial structure (black curve), and remains occluded at the substrate binding site in all structures. These regions are highlighted with grey bands. B. Solvent accessible surface area (SASA) of EC (violet) and IC (green) lumen residues for TM1-TM10 in the initial structure, Model1, and Model2. C. Number of water molecules in the EC and IC halves of the lumen for the initial structure, Model1 and Model2.

The radius profiles (Fig. 5) were calculated using the HOLE program [54], for the starting structure input to TMD (referred to as “Initial”), and averaged over the last 1 ns for 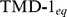 and 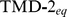. The radii for the part of a monomer between z = 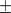
15Å, which approximately defines the upper and lower edges of the lumen, respectively, are shown.

Solvent accessible surface area (SASA) (Fig. 5) was calculated using VMD, for TM1-TM10, and for EL4 and IL1, in the starting structure and in both models. The EC and IC halves were defined as the region between the substrate and the upper or lower edges of the lumen, respectively, i.e, 2
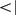
z
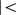
15.

The number of water molecules lying in the EC and IC halves of the lumen of the starting structure and both models (Fig. 5), was calculated between the substrate and the upper or lower edges of the lumen, respectively, i.e., 2 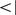
z
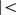
15, to exclude bulk water. In addition, a condition of being within 10 Å of F253, TM1, TM3, TM6, TM8, TM10 or the tip of the EL4 loop (all lining the EC lumen) was also imposed on water molecules in the EC lumen. Similarly, a condition of being within 10 Å of S256, TM1, TM3, TM6, TM8, TM5, or the tip of the IL1 loop was imposed for IC lumen water molecules.

**Figure 6:**
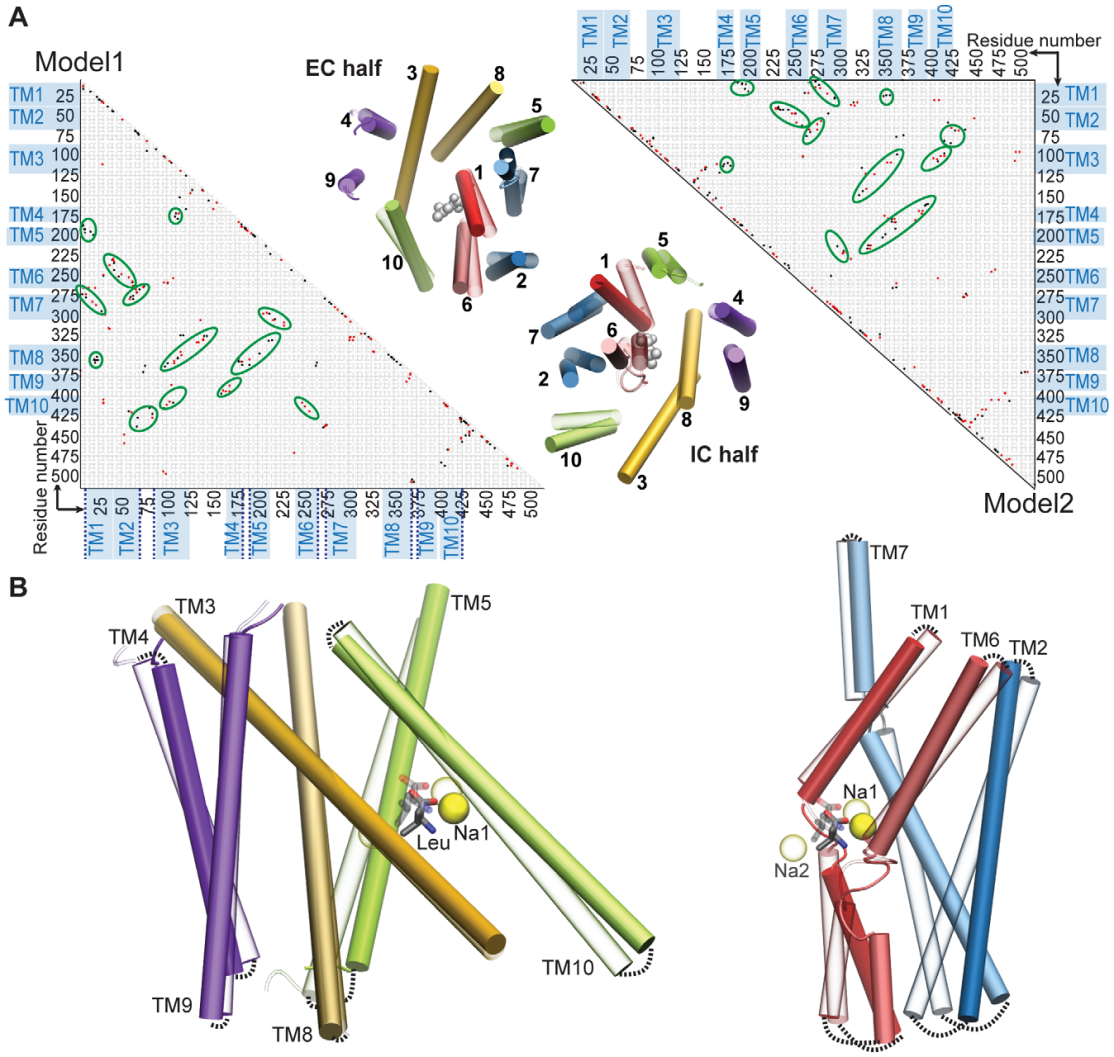
Changes in contacts and conformation in 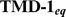 and 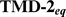 simulations. A. Residue-residue differential contact maps (see Methods) for Model1 (left triangle) and Model2 (right triangle). Contacts broken (black dots) and formed (red dots) are shown. Residue ranges corresponding to TM1-TM10 are marked (blue). The intracellular ends are marked by dotted blue lines in the Model1 map. Regions showing notable contact breakage/formation excluding those beyond TM10 or residues on the diagonal are highlighted (green ovals). Similar trends are observable in 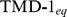 and 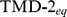. In the center, EC (top) and IC (bottom) views of TM1-TM10 are shown with superimposed snapshots, taken before (transparent) and after (solid) the 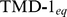 simulation. TM3 and TM8 C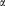 atoms were used for the superposition. Pseudosymmetric pairs of helices are colored the same, with darker colors for TM1-TM5, and lighter for TM6-TM10 i.e. TM1 and TM6 are red, TM2 and TM7 are blue, TM3 and TM8 are golden, TM4 and TM9 are violet, and, TM5 and TM10 are green. Closing of TM1, TM7, and TM10 in the EC half and opening of TM6, TM2, and TM5 in the IC half are clearly visible, corresponding to the differential contact maps. B. Side views of the less mobile TM3-TM4-TM5-TM8-TM9-TM10 scaffold (left) and the highly mobile TM1-TM2-TM6-TM7 bundle (right) are shown separately, in superimposed snapshots taken before (transparent) and after (solid) the 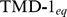 simulation. The coloring scheme is as above. The position of the substrate (gray) and 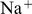 ions (yellow) before (transparent) and after (solid) the simulation are also shown. Dotted black lines highlight the motion in the helices.

The residue-residue differential contact maps for both Model1 and Model2 (Fig. 6) were constructed by determining the contacts “broken” and “formed” in the models, with respect to the starting structure. Contacting residues were determined for any residue, **i**, as residues with heavy atoms within 3.5 Å of **i**, excluding the residues within **i** 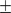
3. Contacts were considered as “broken” when they are present before the simulation but lost in the final model, and “formed” when they are absent before the simulation but appear in the final model.

## Results/Discussion

Here, we describe the features of the LeuT IF state through models generated in this study. We focus on dynamic structural elements and interactions that may play important roles in the OF-to-IF transition and discuss their relevance to the mechanism of LeuT-fold transporters.

### Inward-facing models of LeuT

The method employed for modeling LeuT in the IF state uses a pre-equilibrated crystal structure of LeuT in the OF-occ state, structural information from the vSGLT IF state, and a simulation-based technique that allows the OF structure to dynamically adapt as it is modified to the IF state. This approach involves the study of the possible OF-to-IF transition and IF state dynamics in an environment of explicit lipids, water and ions, which ensures better conservation of protein intramolecular interactions and structure quality as compared to vacuum or dielectric/implicit solvent environments used in standard model-building techniques. Also, only parts of the protein were included in the targeting, leaving the rest of the protein free to respond to the induced conformational change. This is in particular important for conformational adaptation of the side chains and the loops. As in most modeling approaches, this technique is limited by the initial assumptions, particularly that of vSGLT being representative of a possible IF state for the LeuT fold. Our modeling approach, which targets a combination of elements from the LeuT-OF state and a vSGLT-like IF state (pre-Models) rather than directly conforming to the vSGLT structure, mitigates to some degree the possible limitations presented by dissimilarity between LeuT and vSGLT. A major limitation of the presented methodology is associated with the simulation time scale, preventing one from a complete description of the events involved in transport cycle and associated protein conformational changes that occur under experimental or in vivo conditions. This technique may be thus considered as a means of obtaining some structural snapshots that can be scrutinized with experimental techniques to determine their possible role in the transport cycle. Since the methodology only requires information input from the crystal structures of LeuT and vSGLT, that is, it is unbiased by existing knowledge from functional studies of the IF state of LeuT, it is objective in nature, and this allows for the potential extension of this technique to other protein families for which limited structural and functional studies have been reported.

The methodology succeeds in yielding plausible models of the IF state that show consistency with experimental studies, as will be discussed later, thus providing a starting point for further studies about the IF state and the OF-to-IF transition. The substrate remains bound to its binding site throughout 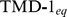 and 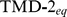, but water starts to directly access the substrate in 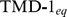, hence Model1 may represent either IF-o, IF-occ, or an intermediate state, while Model2 seems to capture an IF-occ state. We henceforth refer to the models as being in the IF-o/IF-occ or simply, the IF state.

RMSDs of helices TM1-TM10 during the 50 ns TMD+20 ns equilibration in the targeted monomer (monomer A), compared to 70 ns free MD in the second monomer (monomer B), are shown in Fig. 4. Deviations from both the LeuT crystal structure as well as from the starting structure for the TMD simulations (10-ns equilibrated), are shown. These RMSDs represent the overall structural variation in the TM helices forming the inverted repeats, including the EC and IC halves. For both 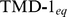 and 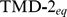, the deviation from the reference structures increases nearly continuously from 0 to 50 ns, as the TMD forces drive the structure from the OF state toward the IF state. After 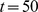 ns, a drop in the deviation occurs, indicating that the structures spring back to a small extent, as is expected at the point where TMD forces are removed. The RMSD soon stabilizes in the following 20 ns of free MD (Fig. 4). The structures clearly relax to a conformation different from the reference as well as from the control OF-occ structures (RMSDs in Table S1, Supporting Information).

Model1 and Model2, the final structures from 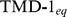 and 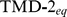 respectively, both show opening in the IC half and further closure on the EC half of the transporter (Fig. 5A), in accordance with proposed sub-states in the alternating-access model [29], [40]. The opening and closure of the lumen are represented by the radius profiles shown in Fig. 5A, calculated for the starting structure and averaged over structures from the last 1 ns of 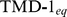 and 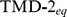. These profiles provide a good overall measure of the opening of the lumen, but may not reflect small crevices in the structure that can accommodate water and ions.

Water penetration defines the location of the EC and IC half-lumens and reveals that both the EC and IC permeation pathways are mainly lined by residues from TM1, TM3, TM6, and TM8 (Fig. 5A). Additionally, TM10 participates in the lining of the EC lumen, while its pseudosymmetry-related helix, TM5, participates in the IC lumen. Accessibility (SASA) of the EC and IC halves of the helices TM1-TM10, and the number of water molecules populating the EC and IC halves of the lumen are presented in Fig. 5B and Fig. 5C, respectively. Among the TM helices lining the EC half of the lumen, the SASA of TM1 does not change appreciably, those for TM3 and TM8 increase, and those for TM6 and TM10 decrease. The SASA increase for TM3 and TM8 can be attributed to the slight tilting of these helices, exposing more residues to the EC solution, and to the substrate shifting towards the IC side, which allows higher accessibility of the adjacent TM3 and TM8 in the EC lumen. The SASA decrease in TM6 and TM10, on the other hand, is due to the narrowing of the EC lumen. The number of water molecules in the EC lumen decreases in both models, consistent with the narrowing of the lumen in the EC half of the transporter (Fig. 5C).

In the IC half, the SASA of TM1, TM6, and TM8 increases dramatically for Model1, while in Model2, only TM6 and TM8 show appreciable SASA increases. High SASA for TM7 in Model1 is due to the formation of a local water-filled cavity, though TM7 stays away from the lumen. Although TM5 participates in the IC lumen, it does not show a large change in SASA, since it moves vertically, moving residues previously accessible to the cytoplasm towards the solvent-accessible IC lumen. Model1 shows a larger extent of water penetration into the IC lumen than Model2, as indicated by SASA and the number of water molecules (Fig. 5). As will be discussed later, one of the bound 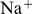 ions is released in 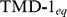 and results in a greater degree of water penetration in Model1 compared to Model2. Model1 may represent the IF state after ion release, while Model2, which retains both bound 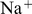 ions, possibly represents an ion-bound IF state. Thus, the observation of a larger extent of water access in the IC lumen of Model1 may be of direct relevance to the transport mechanism, suggesting that ion release allows extensive water filling in the IC pathway which may eventually facilitate the release of the substrate molecule.

Mutagenesis and accessibility studies using MTS reagents have revealed that the cytoplasmic lumen in SERT is lined by residues from TM1, TM5, TM6 and TM8 [18], [20]. The only other reported IF state model of LeuT was shown to be compatible with this accessibility data [20], and was further refined to improve the conformity. Consistent with the experimental data and the previous model, both Model1 and Model2 exhibit the IC lumen as lined by residues from the corresponding helices (TM1, TM5, TM6, TM8, and in addition, TM3) in LeuT. The previous model [20] proposes rigid body rotation of the TM1∶TM2∶TM6∶TM7 bundle as a possible mechanism of transition between the IF and OF states. Model1 and Model2 also capture large structural rearrangements for TM1, TM2, TM6, and TM7. Additionally, due to the dynamical method used to induce the IF state, we are able to describe transitions that are more complex than rigid rotation of the helices, including several changes in interactions, side chain and secondary structural rearrangements and conformational adaptation in several parts of the protein. Previous simulations of substrate pulling on the IC side have described changes in interactions upon the formation of the IF state [24], which are known to be important to transport, from experimental studies [55]. However, more global conformational changes, which are expected to be involved in the formation of the IF state, could not be observed within the simulation time scale (20 ns). In this study, longer simulations (70 ns) and enhanced OF to IF transition have allowed us to capture several functionally relevant interactions and conformational changes, as discussed in the following sections.

### Conformational hotspots in the “LeuT fold”

Residue-residue differential contact maps for the structures before and after 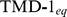 and 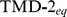 show areas where interactions are lost or newly formed, indicating conformational “hotspots” involved in the transition between the OF and IF states (Fig. 6A). Model1 and Model2 show similar patterns (Fig. 6A) indicating that these changes are not random, but related to the OF-to-IF transition. These changes in contacts, especially among residues belonging to separate TM helices, are thus related to the motion of these elements.

The differential contact maps in Fig. 6A show that in the IF-o/IF-occ state, TM1 loses some contacts with TM5 and TM7 in the IC half, and with TM8 near the center. As is discernible in the top- and side-view snapshots in Fig. 6A and Fig. 6B, this corresponds to the IC-opening motion of TM1. The latter, i.e., the TM1∶TM8 contact variation, is especially interesting since TM1 and TM8 form the Na2 binding site (discussed in the next section). The large TM1 motion observed in the IC half upon inward-opening is consistent with recent MD and FRET studies on the conformational changes involved in IF state formation [25].

TM2 loses contacts with the TM5-TM6 loop, but forms several new interactions with TM6 and TM7 in the IC half, as all three helices curve away to open the IC lumen. TM3 and TM8 are cradled by the V-shaped TM4∶TM5 pair in the EC half and the TM9∶TM10 pair in the IC half (Figs. 6A and 6B). Due to the transition, TM3 forms new contacts with TM8, as TM8 slants towards it to facilitate the opening of the IC lumen. Thus, TM8 loses contacts with the TM4∶TM5 IC end, while also pushing TM3 to form new interactions with TM9∶TM10.

Among other notable changes are the gain in interactions between TM4 and TM9, between TM5 and TM7, and between the TM5-TM6 EC loop and TM7 (Fig. 6A). Interestingly, among these elements, only TM5 is directly involved in the lumen. It is possible that newly formed interactions with TM7 may play a favorable role in the vertical movement of TM5, which brings the initially distant residue E192 close to the IC lumen. The significance of this motion is discussed in the next section. Taken together, these results suggest that all 10 helices of the LeuT fold, as well as several intermediate loop regions, participate in the conformational changes involved in the OF-to-IF transition observed in 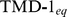 and 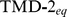.

**Figure 7:**
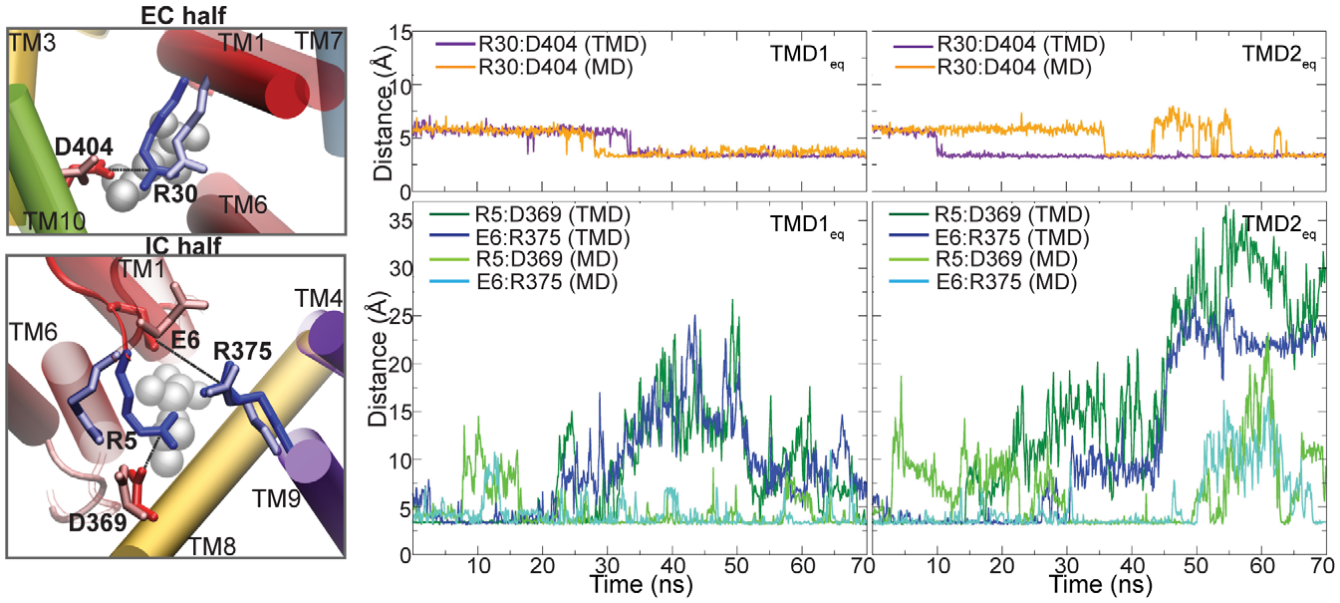
Variation in salt bridge interactions in the EC and IC halves of the lumen. Snapshots of salt bridges in the EC (top) and IC (bottom) half-lumens before (light) and after (dark) the 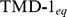 simulation. The coloring scheme is as in Fig. 6. Variation of distance between salt bridging residues in the EC (top) and IC (bottom) half-lumens for 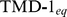 (left) and 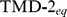 (right) are shown. The distances are compared between monomer A, which undergoes TMD and monomer B, which undergoes free MD and serves as a control system.

We also monitored the change in salt bridge interactions across the lumen of the putative substrate transport pathway in the LeuT crystal structure. We found three interesting cases, of which two salt bridges, R30∶D404 and R5∶D369, are formed by conserved residues in the NSS family [3] and lie in the EC and IC halves, respectively. The third salt bridge, E6∶R375, is also observed in the IC half, nearly parallel to the R5∶D369 bridge. The R30∶D404 interaction was observed to be water-mediated in the OF-occ LeuT crystal structure [2], but several subsequent inhibitor-bound structures showed direct salt bridge formation for this pair [7], [8]. Fig. 7 shows the variation in distance between salt bridging residues for 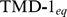 and 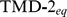 simulations, in the monomer undergoing TMD, as well as the second monomer undergoing free MD (control system). In both 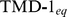 and 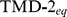 simulations, we observe that the R30∶D404 pair transits from a water-mediated to a direct salt bridge interaction when the structure transits towards the IF-o/IF-occ state. However, the control system also shows direct salt bridge formation for R30∶D404, albeit with larger fluctuations. This suggests that in the OF state, the R30∶D404 salt bridge is a fluctuating interaction and may switch from water-mediated to a direct one, and back. However, the IF state clearly favors a direct R30∶D404 salt bridge. Similarly, on the IC side, R5∶D369 and E6∶R375 also show fluctuations in the control system, but show a much larger degree of fluctuation and spend much more time in the broken state in the 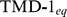 and 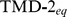 simulations. Clearly, the IF-o/IF-occ state favors breakage of R5∶D369 and E6∶R375 salt bridges in the IC half. Notably, the R5∶D369 pair in LeuT is equivalent to the R60∶D436 in DAT, which is part of an intracellular interaction network involved in regulating access of the substrate to the cytoplasm [55]. Also, substrate pulling toward the cytoplasmic side is reported to be associated with rearrangement of R5 and D369 [24]. Since these IC salt bridges do not form a complete IC plug, they do not appear to strongly affect water penetration, but they may be expected to play a role when the larger substrate molecule moves across this region. The importance of salt bridge rearrangements in transporter mechanism have earlier been highlighted in simulation studies, e.g, for the ATP/ADP carrier [56] and glycerol-3-phosphate transporter [57]. The favoring of R30∶D404 salt bridge formation in the EC lumen, and R5∶D369 and E6∶R375 salt bridge breakage in the IC lumen thus suggest that these may play a role in the narrowing of the EC lumen and the widening of the IC one.

### Release of Na2 upon OF-to-IF transition

LeuT transports amino acids by using energy derived from coupling with 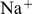 transport. The binding sites for these functionally critical 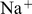 ions were revealed in the LeuT crystal structure, which reported two bound 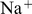 ions, named as “Na1” and “Na2” [2]. Na1 is coordinated by leucine, the substrate, itself, along with residues from TM1, TM6, and TM7. Na2 is 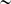
6Å away from the C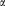 of the substrate and is coordinated by residues from TM1 and TM8.

**Figure 8:**
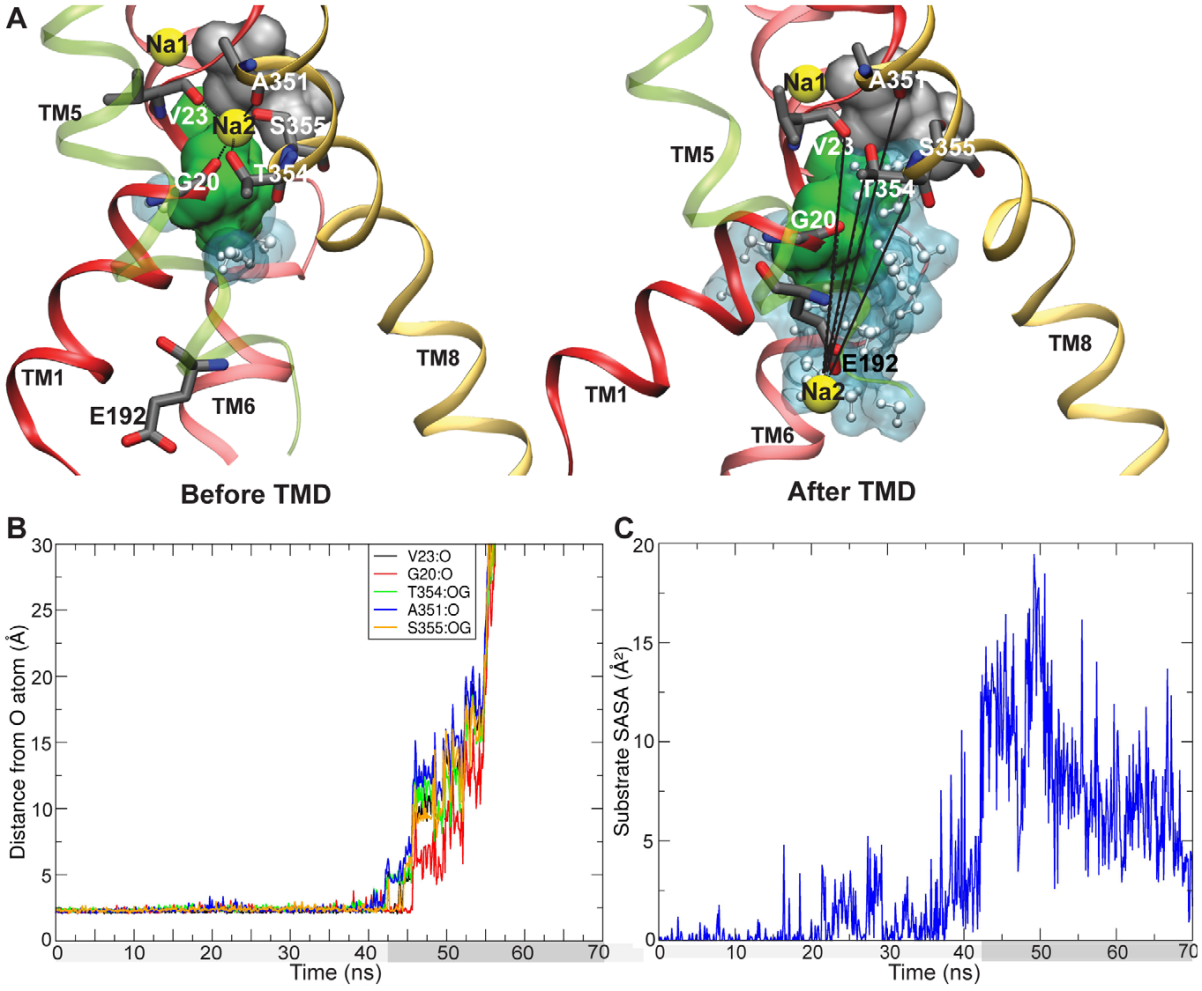
Na2 release and water access to the substrate in the 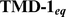 simulation. A. The Na2 binding site before (left) and after (right) 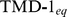 is shown, with the Na2 binding residues marked. E192, which binds Na2 (yellow) along its unbinding and release from the binding site is also shown. The substrate (Leu) is shown in gray. N21 and S256, which protect the substrate from water within a cavity below the substrate, are shown as a green surface. TM1 (red) and TM8 (golden), which bind Na2, TM6 (light red) which carries S256, and TM5 (green, transparent), which carries E192 are shown. Other parts of the protein are hidden for clarity. Water is shown as white molecules with a blue surface. B. The distance of Na2 from O atoms of binding residues is shown for the 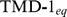 simulation. Na2 release occurs around 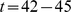 ns. C. SASA of the substrate is shown for the 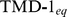 simulation. The substrate SASA increases around the same time as Na2 release occurs, due to water access through the empty Na2 site.

One of the most interesting occurrences during the TMD simulations was the release of Na2 in 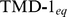, as the OF-to-IF transition proceeded (Fig. 8A). Na2 is coordinated by backbone and side-chain oxygen atoms from TM1 (G20, V23) and TM8 (A351, T354, S355) residues. The Na2-binding residue distances plotted over the transition trajectory indicate that the release occurs after nearly 40 ns of TMD (Fig. 8B), when the distance between TM1 and TM8 has sufficiently increased.

This release of Na2 is analogous to the 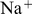 release reported recently in simulations of IF vSGLT [58] as well as for Mhp1 [33]. We also observe that E192 coordinates Na2 as it is released, and appears to escort it out of the protein. Notably, E192 of LeuT is at the same position as D189 of vSGLT (Fig. 3), a residue observed to participate in Na2 release in vSGLT [58], and also shown to be important for function in vSGLT homologs [59], [60]. It is also interesting that E192 becomes proximal to the IC lumen (and hence accessible to 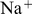) only after the vertical movement of TM5 during 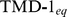.

We also noted that Na2 release does not occur in 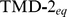. On comparison of 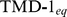 and 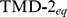, we observe that though loss in TM1∶TM8 interactions occurs in both simulations (Fig. 6A), the movement of TM1 with respect to TM8 is larger in 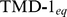. This slight difference appears to suffice for Na2 release, thus indicating that TM1 motion plays a critical role in Na2 release.

A second interesting feature associated with Na2 release is that after Na2 dislodges from its binding site, water starts to access the substrate from the side of the now-empty Na2 binding site (Fig. 8A). The N21-S256 interaction pair, shown as a green surface in Fig. 8A protects the substrate from water filling in another cavity below it, thus the empty Na2 binding site provides the only immediate access point to the substrate. The opening of a water-accessible pathway to the substrate translates into a sudden increase in SASA of the substrate observed around the same time as Na2 is released (Fig. 8B). Notably, in this state, the substrate while being directly accessible to water in the IC lumen, is only separated from water in the EC lumen by the Y108-F253 hydrophobic plug. It thus appears that a slight rotation of either the Y108 or F253 side chains may allow water and ions from the EC lumen to access the substrate binding site and the IC lumen in the IF state. Such a structural arrangement could explain substrate-dependent channel-like behavior reported for several NSS family members, including DAT-1 [61], NET [62], GAT-1 [63] and SERT [64].

The behavior of Na2 is especially relevant in the context of the LeuT-fold transporters. Three other transporters adopting the LeuT fold, vSGLT, Mhp1, and BetP, are proposed to have a 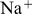 ion bound at the site analogous to the Na2 binding site in LeuT [31], [32], [34]. 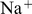 is necessary for the function of NSS members as well as several other LeuT fold transporters, but the mechanism of its binding and release is not known. Thus, the observation that Na2 is released only upon TM1 motion, and that its unbinding allows water access to the substrate from the intracellular side, possibly describe critical steps in the transport mechanism.

### Coupling between inverted repeat units

As discussed in Methods, the IF state modeling was achieved by inducing OF-to-IF transition using two TMDs, each 50 ns long, which were followed by 20 ns of free MD. In 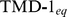, only the TM1-TM5 fold was targeted, i.e., conformational change was induced by addition of external forces to only the TM1-TM5 fold. In 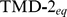, TM1 and TM6 were excluded from targeting while the remaining eight helices of the inverted repeat were targeted. Both the 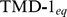 and 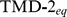 exhibited inward-opening with outward-closure. Parts of the protein that are not targeted, including the connecting loops and helices as well as TM11 and TM12, respond naturally under equilibrium MD conditions, to the structural changes induced in the targeted region and participate in the conformational changes that lead to the IF-o/IF-occ state.

Interestingly, even in 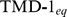 where only one of the pseudosymmetric domains is targeted, LeuT exhibits transition to the IF state (Fig. 6). That is, while the change is forced in only half of the inverted repeat, the other half responds and participates in the transition. This suggests that conformational change in one of the two inverted repeat units may be sufficient to induce a transition in the rest of the protein. Though we attempted to confirm this behavior with the second fold (TM6 -to- TM10), the major difference in TM6 (relative to TM1) orientation among vSGLT and LeuT did not allow successful modeling, as discussed in Methods. Based on the observation of the formation of the IF state, we hypothesize about two important features of the LeuT transporter mechanism: first, the intimate association between the two pseudosymmetric domains extends beyond structural association, to functional cooperation, and second, the two folds may act as highly coupled functional units, where perturbations in one can induce conformational change in the other.

Similar observations were made in 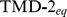. TM1 and TM6 form the functional core of the protein, participating in the substrate and 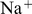 binding sites, and are expected to play a primary role in substrate translocation. One would thus expect that their exclusion from targeting forces would inhibit the transition to an IF state. It is, thus, initially surprising to note inward-opening in the 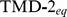 simulation. However, on closer examination, we note that TM1 and TM6 respond to the conformational change in the surrounding helices, and show a corresponding motion, which contributes to inward-opening.

### TM2 and TM7 facilitate motion in TM1 and TM6

TM2 and TM7 brace TM6 and TM1, respectively. They are placed in positions away from the substrate binding or translocation pathway, and do not appear to be involved in these processes. We do observe, though, that TM2 and TM7 show relatively large displacements that could induce motion in the core helices, TM6 and TM1.

Fig. 6A and Fig. 6B show snapshots of these helices before and after the OF-to-IF transition. TM1, TM7, TM2, and TM6 show a lumen-closing motion on the EC half and a lumen-opening motion on the IC half. Notably, these EC-closing and IC-opening motions are visible in both 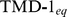 and 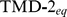 where forces were applied to TM1 but not to TM6 in the former, and on neither TM1 nor TM6 in the latter. Thus, it is apparent that the motions seen in TM1 and TM6 are induced through their coupling to the surrounding helices. Considering the arrangement of TM2 and TM7 with respect to TM6 and TM1, it appears that these assume a functional role of inducing or facilitating TM6/TM1 motion associated with lumen opening or closure.

Experimental cysteine-scanning studies of TM2 and TM7 residues in the NSS member, SERT, have shown that both are mostly inaccessible to MTS reagents due to low solvent accessibility, suggesting no participation in the lumen. Yet, these helices are known to be important for transport function, and contain critical residues whose mutations strongly affect transporter activity [65], [66]. Five residues forming a “critical stripe” on TM7 were proposed to be involved in conformational changes induced by 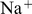 binding, and in transporter function [66]. These were N368, F373, F377, F380, and Y385 in SERT, which correspond to N286, V291, G295, S298, and V303 in LeuT. Of these, three interact with TM1, one of which also coordinates Na1. This TM1∶TM7 interaction was maintained during the simulations, while TM7 and TM1 moved together. Thus, while TM2 and TM7 do not participate directly in the lumen, as suggested by mutagenesis and crystal structures, their criticality can be explained by their role as facilitators of TM1 and TM6 motion.

A previous study provided a model for alternating access where the TM1∶TM2∶TM6∶TM7 bundle rotates as a rigid body, allowing alternate OF and IF states [20], [30]. Our simulations also identify roles for TM2 and TM7 in the alternating access mechanism. However, we propose that TM1∶TM2∶TM6∶TM7 do not behave as a rigid body. The extent of TM1∶TM7 and TM6∶TM2 motion in the simulations differs. We also observe that TM1∶TM7 motion appears more important for the EC closure while TM2∶TM6 motion appears more relevant to IC opening, thus suggesting two interrelated roles for these pairs of helices.

### Concluding remarks

We have employed molecular dynamics in combination with structure-based threading and homology modeling to construct an atomic model for the IF state of LeuT and to investigate some of the structural and dynamical elements that are involved in the OF-to-IF transition. The modeling methodology developed here can be extended to obtain models for, or study transition between structurally unknown states in other proteins.

Incorporating dynamics in the method has resulted in revelation of novel functionally-relevant features of “LeuT-fold” transporters. TM1, TM3, TM6, and TM8, along with TM10/TM5 residues line the lumen. Putative substrate and 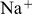 release pathways are revealed either indirectly based on the calculated water occupancy profiles, or directly upon the captured unbinding events. The structural elements involved in the alternation between states are also described. Though initially one might expect these elements to only involve helices participating directly in the lumen, the present study suggests that additional elements, namely, TM2 and TM7, may also play a critical role in alternating access, in this case by facilitating the movement of TM1 and TM6. An interesting revelation is the conformational coupling of the symmetry-related subunits in LeuT, and the suggestion that they could show symmetry in function. While hints of such behavior are already provided by the alternate participation of symmetry-related TM10 and TM5 in the EC and IC halves of the lumen, stronger evidence is obtained from one of the simulations (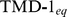) where structural modification of only one of the two domains induced an overall transition to the IF state. This observation suggests that each of the two pseudosymmetric domains may represent a functional unit capable of inducing transition in the full protein. Several observations reported here are consistent with experimental studies, though further experimental evidence would be required to test some of the novel hypotheses developed in this study.

## Supporting Information

Dataset S1 PDB format structure file for Model1.
(0.66 MB TXT)

Dataset S2 PDB format structure file for Model2.
(0.66 MB TXT)

Figure S1 Comparison of pre-TMD and TMD targets with the LeuT crystal structure. The pre-target, pre-Model1 and pre-Model2 structures (in solid colors) superimposed on the LeuT crystal structure (transparent) are shown. TM10 is hidden for clarity. Pseudosymmetric pairs of helices are colored the same, with darker colors for TM1 to TM5, and lighter for TM6 to TM10 i.e. TM1 and TM6 are red, TM2 and TM7 are blue, TM3 and TM8 are golden, TM4 and TM9 are violet, and, TM5 and TM10 are green. The intermediate targets used in the modeling methodology are clearly more open on the intracellular side, compared to the OF-occ LeuT crystal structure. Substrate (gray) and Na^+^ ions (yellow) are also shown for pre-Model1 and pre-Model2.
(3.06 MB TIF)

Table S1 Key RMSDs of Cα atoms in the starting, intermediate and final structures.
(0.05 MB PDF)

Video S1 Model1 generation. 70 ns MD simulation trajectory of transition from OF state to Model1 IF state.
(9.12 MB MOV)

Video S2 Model2 generation. 70 ns MD simulation trajectory of transition from OF state to Model2 IF state.
(9.65 MB MOV)
