# Supplementary figures and images for: Modeling and Dynamics of the Inward-Facing State of a Na+/Cl− Dependent Neurotransmitter Transporter Homologue
Source: PLoS Comput Biol. 2010 Aug 26;6(8):e1000905. doi: 10.1371/journal.pcbi.1000905 (PMC2928745; doi:10.1371/journal.pcbi.1000905)

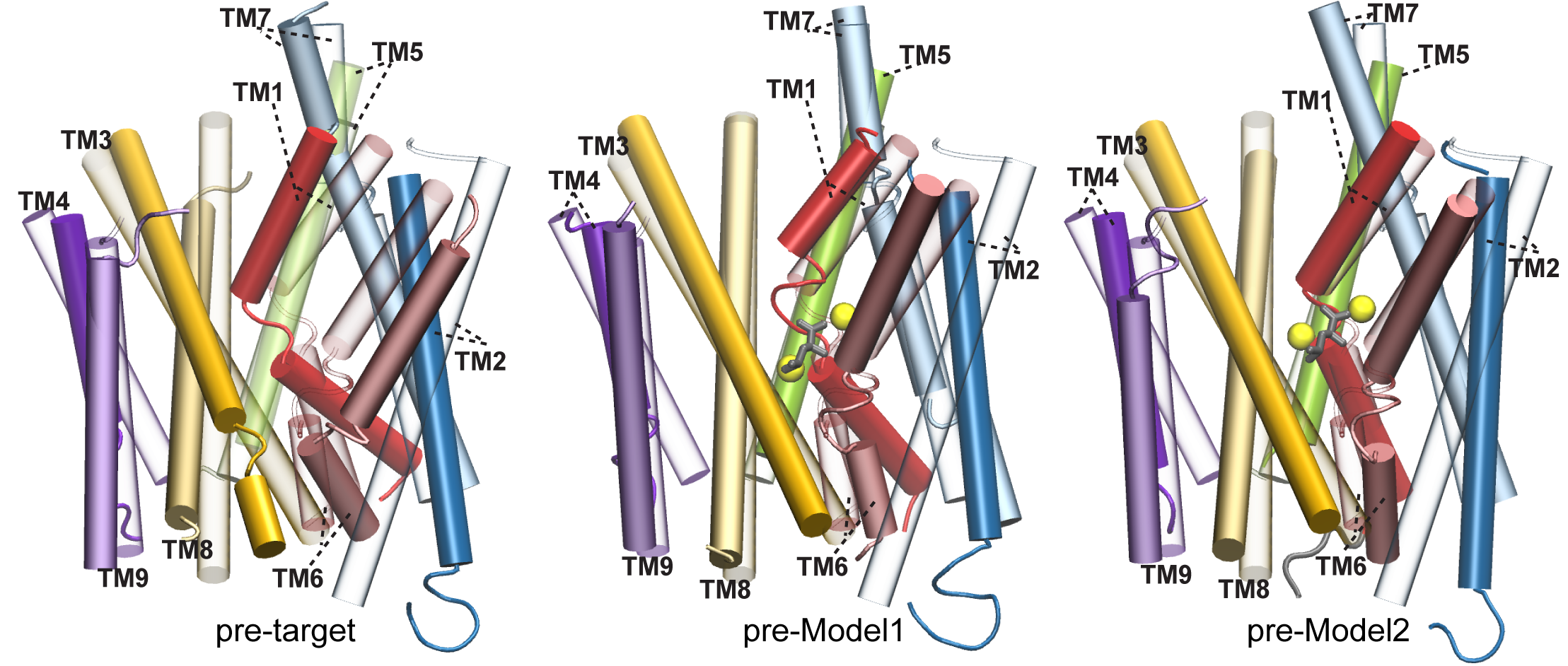

Supplement: Figure S1 — Comparison of pre-TMD and TMD targets with the LeuT crystal structure. The pre-target, pre-Model1 and pre-Model2 structures (in solid colors) superimposed on the LeuT crystal structure (transparent) are shown. TM10 is hidden for clarity. Pseudosymmetric pairs of helices are colored the same, with darker colors for TM1 to TM5, and lighter for TM6 to TM10 i.e. TM1 and TM6 are red, TM2 and TM7 are blue, TM3 and TM8 are golden, TM4 and TM9 are violet, and, TM5 and TM10 are green. The intermediate targets used in the modeling methodology are clearly more open on the intracellular side, compared to the OF-occ LeuT crystal structure. Substrate (gray) and Na+ ions (yellow) are also shown for pre-Model1 and pre-Model2. (3.06 MB TIF) [file pcbi.1000905.s003.tif]
