# Supplementary material for: Modeling and Dynamics of the Inward-Facing State of a Na+/Cl− Dependent Neurotransmitter Transporter Homologue
Source: PLoS Comput Biol. 2010 Aug 26;6(8):e1000905. doi: 10.1371/journal.pcbi.1000905 (PMC2928745; doi:10.1371/journal.pcbi.1000905)
Supplement: Table S1 — Key RMSDs of Cα atoms in the starting, intermediate and final structures. (0.05 MB PDF) [file pcbi.1000905.s004.pdf]

Structures	All C_α	TM1-TM10 C_α
$LeuT_0$: $LeuT_{10}$	1.3	0.9
$LeuT_0$:vSGLT ; $LeuT_{10}$:vSGLT	-	5.1 ; 5.0
pre-target:vSGLT	-	0.9
pre-target†:$LeuT_0$; pre-target:$LeuT_{10}$	15.5 ; 15.5	5.4, 5.3
pre-Model1:vSGLT	-	3.9
pre-Model1:$LeuT_0$; pre-Model1:$LeuT_{10}$	3.5 ; 3.2	3.6 ; 3.4
pre-Model2:vSGLT	-	3.3
pre-Model2:$LeuT_0$; pre-Model2:$LeuT_{10}$	3.6 ; 3.5	3.7 ; 3.5
Model1:$LeuT_0$; Model1:$LeuT_{10}$	2.9 ; 2.7	2.6 ; 2.3
Model2:$LeuT_0$; Model2:$LeuT_{10}$	3.1 ; 2.9	2.5 ; 2.2
Model1:pre-Model1	2.7	2.6
Model2:pre-Model2	2.9	2.6
Model1:Model2	2.6	2.1
Model1:Control1	2.9	2.4
Model2:Control2	3.1	2.3
Control1:$LeuT_0$; Control1:$LeuT_{10}$	1.7 ; 1.7	1.0 ; 0.9
Control2:$LeuT_0$; Control2:$LeuT_{10}$	1.3 ; 1.4	0.9 ; 0.8

$LeuT_0$: LeuT crystal structure PDB code 2A65 (reported in the OF state), $LeuT_{10}$: LeuT OF state equilibrated for 10 ns, used as the starting structure for the TMDs, vSGLT: vSGLT crystal structure PDB code 3DH4, pre-target: Structure used as the target for pre-TMDs, obtained after threading of the LeuT sequence on the vSGLT structure using the structure-based alignment, pre-Model1 and pre-Model2: LeuT monomer A after pre-TMDs where TM1-TM5 and TM2-TM5 + TM7-TM10 were included in targeting, respectively- these serve as targets for TMD-1_{eq} and TMD-2_{eq}, Model1 and Model2: LeuT monomer A after TMD-1_{eq} and TMD-2_{eq}, respectively. Control1 and Control2: LeuT monomer B after 70 ns of MD where monomer A was subjected to TMD with TMD-1_{eq} and TMD-2_{eq}, respectively. RMSDs from vSGLT are not reported for the full monomer, since LeuT and vSGLT have structural similarity only in TM1 to TM10.

†Structure obtained after threading of LeuT on vSGLT. Apart from TM1 to TM10, most other secondary structural elements of LeuT and vSGLT do not correspond hence are incorrectly modeled, reflected in the high RMSD for all C_α and lower RMSD for TM1-TM10 C_α. Thus, TMDs were adopted to correct this issue (see pre-Models and Models).
